# Supplementary material for: Minimally Invasive Free-Breathing Gating-Free Extracellular Cellular Volume Quantification for Repetitive Myocardial Fibrosis Evaluation in Rodents
Source: Biomolecules. 2025 Dec 12;15(12):1732. doi: 10.3390/biom15121732 (PMC12731170; doi:10.3390/biom15121732)
Supplement: Supplementary file 1 [file biomolecules-15-01732-s001.zip › biomolecules-3983967-supplementary.pdf]

Supplemental Figures and Data

Normality Testing

Metric	LV Pre-R_1	LV Post-R_1	LV ΔR_1	LV Blood Pool ΔR_1	Jugular Vein ΔR_1	LV ECV Scan 1	LV ECV Scan 2	JV ECV Scan 1	JV ECV Scan 2
Normality Test					Result				
D'Agnostio & Pearson	Failed	Failed	Passed	Passed	Passed	Passed	Failed	Passed	Passed
Shapiro-Wilk	Failed	Failed	Failed	Passed	Passed	Passed	Passed	Passed	Passed

Supplemental Table S1. Comprehensive summary of normality testing for all metrics evaluated as part of the study. The left ventricle Pre-R1, Post-R1 and DeltaR1 values failed 5/6 tests for normality and thus the median and IQR are utilized to describe the dataset. The LV blood pool and JV blood sources for ECV normalization did pass all four normality tests and thus the mean and standard deviation are used to summarize the dataset. For all ECV values from each scan and for each blood source, only the D'Agnostio & Pearson test for second scan utilizing LV blood pool normalization reported a non-normal distribution. Qualitative review of this datasets Q-Q plot deemed it sufficiently normal to utilize a parametric t-test for comparison as well as the mean and standard deviation sufficient to summarize the dataset.
